# Late-onset sepsis in newborns caused by *Bacillus Cereus*: a case report and literature review

**DOI:** 10.1186/s12941-024-00712-4

**Published:** 2024-07-26

**Authors:** Wang Zhang, Caihua Ma, Linghui Hu, Ling Wang, Falin Xu

**Affiliations:** The Third Affiliated Hospital of Zhengzhou University, No. 7, Kangfuqian Street, Erqi District, Zhengzhou, Henan 450052 China

**Keywords:** *Bacillus Cereus*, Neonates, Sepsis, Mortality rate, Antimicrobial therapy, Infection control

## Abstract

*Bacillus cereus* is a bacterium capable of causing late-onset neonatal sepsis. By analyzing 11 cases, this study investigates the diagnosis, treatment, and prognosis of *Bacillus cereus* infections, aiming to provide insights into clinical diagnosis and therapy. The study scrutinized 11 instances of late-onset neonatal sepsis, including two fatalities attributable to *Bacillus cereus*, one accompanied by cerebral hemorrhage. An examination and analysis of these cases’ symptoms, signs, laboratory tests, and treatment processes, along with a review of related literature from 2010 to 2020, revealed a high mortality rate of 41.38% in non-gastrointestinal infections caused by *Bacillus cereus*. Our findings underscore the critical importance of rapid diagnosis and effective antimicrobial therapy in reducing mortality rates. Once the source of infection is identified, implementing effective infection control measures is essential.

**Supplementary Information:**

The online version contains supplementary material available at 10.1186/s12941-024-00712-4.

## Highlights

This study offers the first in-depth analysis of late-onset neonatal sepsis caused by *Bacillus cereus*.

It reveals a high mortality rate associated with *Bacillus cereus* infections in neonates.

The research uncovers potential links between late-onset sepsis and cerebral hemorrhage.

The importance of timely diagnosis and antimicrobial therapy is emphasized.

New strategies and perspectives for preventing and controlling *Bacillus cereus* infections are presented.

**Supplementary Information:**

The online version contains supplementary material available at 10.1186/s12941-024-00712-4.

## Introduction

Neonatal sepsis is a critical clinical emergency, particularly in newborns, where the condition often deteriorates rapidly, posing a severe threat to life [1]. Sepsis is relatively high in neonates, who are more susceptible to infections due to their underdeveloped immune systems [2]. Common pathogens include a range of bacteria, viruses, and fungi, with bacteria being the most frequent causative agents [3]. However, neonatal sepsis caused by *Bacillus cereus* is relatively rare in clinical settings, complicating diagnosis and treatment [4]. *Bacillus cereus*, a bacterium widely distributed in the environment, can survive in diverse conditions [5]. While commonly associated with food poisoning, it can also cause severe non-gastrointestinal infections, particularly in immunocompromised newborns [6].

*Bacillus cereus*, a Gram-positive, spore-forming bacterium, is ubiquitously found in soil, water, air, and food. Its heat-resistant spores enable survival in extreme environments and potential transmission through the food chain [7]. Although primarily associated with food poisoning, *Bacillus cereus* can cause severe systemic infections, such as sepsis, meningitis, and pneumonia [8]. In newborns, infections caused by *Bacillus cereus* are particularly hazardous due to their immature immune systems, making them more vulnerable to invasive pathogens [9]. Furthermore, *Bacillus cereus* infections may be misdiagnosed or delayed due to atypical clinical presentations [10]. Thus, a comprehensive understanding of *Bacillus cereus* and its infection characteristics in newborns is crucial for improving clinical diagnosis and treatment.

Previous research has primarily focused on foodborne illnesses caused by *Bacillus cereus*, with fewer studies on systemic infections, especially in newborns. Existing literature indicates that although these infections are rare in neonates, their consequences are often severe [8]. Previous studies have also highlighted diagnostic challenges with *Bacillus cereus* infections, such as conventional blood cultures failing to detect the bacterium or it being mistaken as a contaminant due to its environmental ubiquity [8, 11, 12]. Additionally, uncertainties remain in clinical management and treatment strategies for *Bacillus cereus* infections. Therefore, more in-depth research on neonatal sepsis caused by *Bacillus cereus* is vital for enhancing diagnostic accuracy and treatment effectiveness.

Infections caused by *Bacillus cereus* in newborns usually present non-specific clinical symptoms, such as fever, respiratory distress, and poor feeding, which are common in neonates and increase the risk of misdiagnosis [8]. Moreover, some strains of *Bacillus cereus* exhibit natural or acquired resistance to commonly used antibiotics, complicating treatment [13, 14]. Although some studies have proposed treatment recommendations for these infections, their efficacy and universality still need to be validated due to the limited number of cases. Therefore, comprehensive and systematic research is necessary to provide clinicians with more accurate diagnostic guidelines and effective treatment options.

This study aims to comprehensively analyze the diagnosis and treatment process of late-onset neonatal sepsis caused by *Bacillus cereus*. We synthesized information from 11 relevant studies [15–25] and eleven case reports from our institution. We have compiled detailed data on 58 cases of Bacillus cereus infection in neonates and aim to conduct in-depth research through these eleven cases of late-onset neonatal sepsis. We seek to reveal the characteristics and challenges of this rare but serious infection.Specifically, our focus includes the clinical manifestations of *Bacillus cereus* infection, diagnostic difficulties, treatment options, and prognostic factors. Additionally, by reviewing related literature over the past decade, we aim to explore the epidemiological trends and patterns of *Bacillus cereus* infections. This research will enhance medical professionals’ understanding of neonatal sepsis caused by *Bacillus cereus* and aid in developing more effective prevention and treatment strategies. This, in turn, provides a stronger scientific foundation and methodological guidance for the clinical treatment of neonatal sepsis.

## Method

We conducted a comprehensive review of 11 relevant studies [15–25] and 11 case reports from our institution, summarizing detailed data on 58 cases of Bacillus cereus infection in newborns. Through an in-depth investigation of 11 cases of late-onset neonatal sepsis caused by B. cereus, we identified two typical cases in which newborns succumbed to systemic bacterial infections triggered by B. cereus. This highlights the fatal nature of systemic sepsis due to B. cereus in young infants early in life. These 11 cases occurred between 2010 and 2020 at the Level III neonatal intensive care unit of the Third Affiliated Hospital of Zhengzhou University. The term “late-onset neonatal sepsis” mentioned in this paper is defined based on the preliminary guidelines for the diagnosis and treatment of neonatal sepsis established by the Neonatology Group of the Chinese Pediatric Society. The main criteria include sepsis, a syndrome of systemic inflammatory response caused by various pathogens (including bacteria, viruses, and protozoa) with the identification of pathogenic bacteria (including bacteria and fungi), termed septicemia. Neonatal sepsis is classified as early-onset sepsis (EOS) and late-onset sepsis (LOS) based on the onset time, with EOS typically occurring at ≤ 3 days of age, and LOS generally occurring > 3 days of age [26].

Regarding the identification of Bacillus cereus, the method follows the national standard (GB/T 4789.14–2014) coupled with biochemical identification and testing using the VITEK2 automatic bacterial identification system. The antibiotic susceptibility screening in the cases is as follows: resistance is indicated when the minimum inhibitory concentration (MIC) of penicillin is > 32, ceftriaxone > 32, meropenem = 0.19 is sensitive, vancomycin < 4 is sensitive, tetracycline = 4 is sensitive, erythromycin = 0.25 is sensitive, and cefotetan > 256 is resistant.

## Case report

### Case 1

**Fig. 1:**
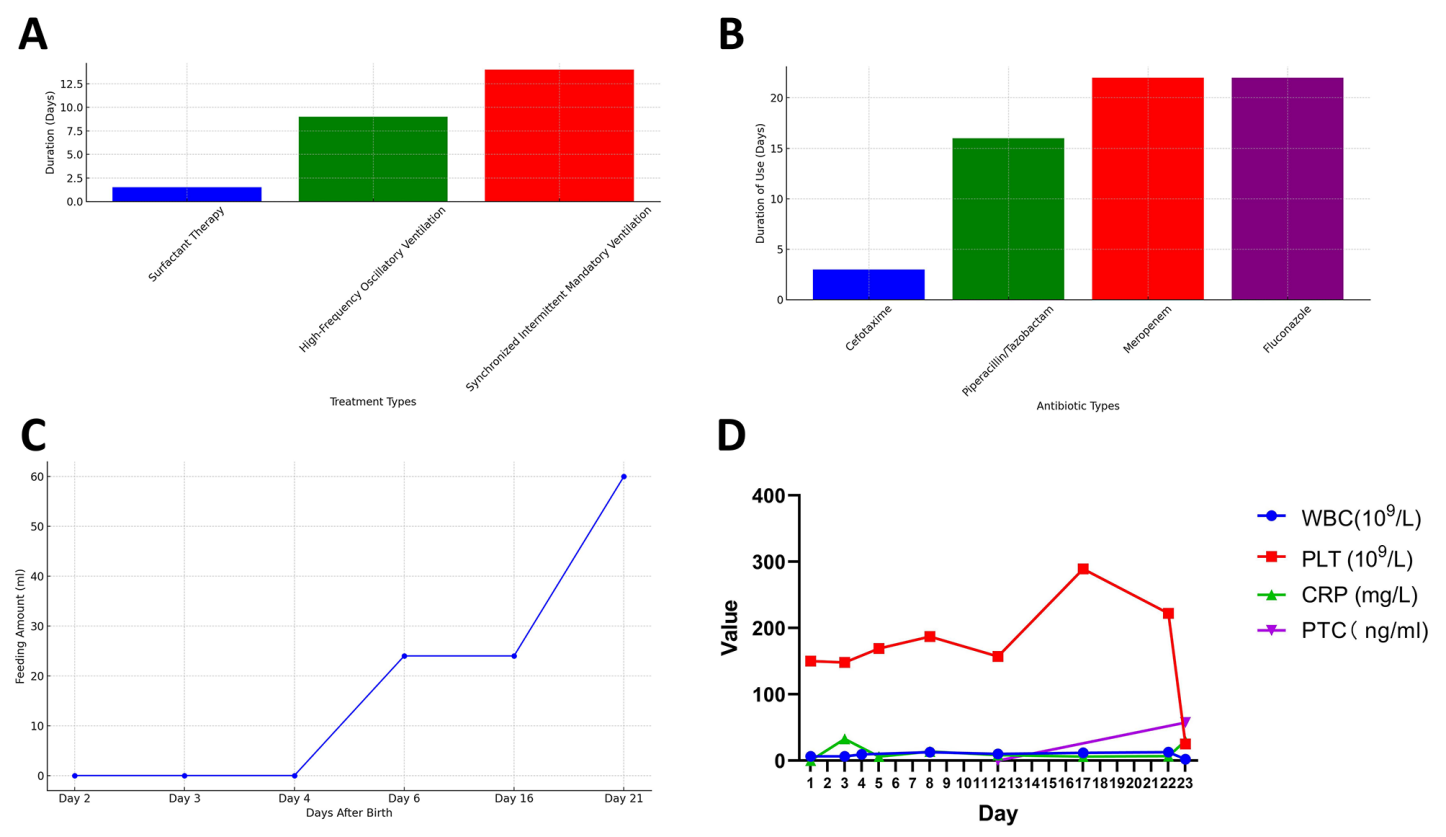
The clinical course of case 1 with late-onset *Bacillus cereus* sepsis. Note: **(A)** Detailed information on respiratory support therapy (duration and effects of poractant alfa treatment, high-frequency oscillatory ventilation, and synchronized intermittent mandatory ventilation); **(B)** Antibiotic treatment history (timing and effects of cefotaxime (0.04 g, q12h), piperacillin/tazobactam (0.09 g, q12h), meropenem (0.04 g), and fluconazole (13 mg)); **(C)** Feeding process (initiation time of feeding, suspension of feeding, increase in feeding volume, and its impact on the patient); **(D)** Laboratory test results from birth to day 22 (changes in WBC, PLT, CRP, PCT values)

A male neonate, the third child from a fourth pregnancy, was born at 27 + 2 weeks gestation, weighing 940 g. The 38-year-old mother delivered via cesarean section due to hemolysis, elevated liver enzymes, and lowplatelets syndrome (HELLP) syndrome, chronic hypertension, and preeclampsia. Figure 1 illustrates the clinical course and diagnostic indicators from admission to day 22 of life.

The neonate scored 8 in both 1-minute and 5-minute Apgar assessments. He was admitted to the neonatal intensive care unit (NICU) due to prematurity, meager birth weight, and neonatal respiratory distress syndrome. Within 1.5 h post-birth, he received two doses of protectant alfa (200 mg/kg), followed by 9 days of high-frequency oscillatory ventilation and 14 days of synchronized intermittent mandatory ventilation. Due to high-risk factors for maternal infection, he was treated empirically with cefotaxime (0.04 g, q12h) for the first three days post-birth. Following abnormal complete blood counts and elevated C-reactive protein (CRP) levels (White blood cell count (WBC) 6.39 × 10^9^/L, refer: 10 × 10^9^~26 × 10^9^/L; CRP 32.79 mg/L, refer: 0∼8 mg/L), his antimicrobial therapy was switched to piperacillin/tazobactam (0.09 g, q12h). Infection indicators were monitored and appeared normal, leading to the cessation of antibiotics on day 19.

Feeding with minimal volumes of expressed breast milk commenced on day 2 but was temporarily halted on day 3 due to abdominal distension. On day 4, the neonate expelled 2 ml of hemorrhagic fluid from the tracheal tube. Fine crackles were audible in both lungs, and the bleeding was managed with hemostatic drugs and coagulation factors without further incidents. Feeding was resumed on day 6 and gradually increased to 24 ml by day 16, with good tolerance. By day 21, feeding reached 60 ml every two hours via nasogastric tube. Cranial ultrasounds showed no significant abnormalities, cardiac functions were normal, and oxygen therapy was progressively reduced to high-flow oxygen therapy. From day 1 to day 7, an umbilical venous catheter was used, which was replaced by a peripherally inserted central catheter (PICC) from day 7 to day 22.

On day 22, the infant’s condition deteriorated rapidly, exhibiting frequent apnea, a drop in transcutaneous oxygen saturation to 60%, cyanosis, poor responsiveness, and bradycardia. His bloodwork showed a decrease in WBC (2.26 × 10^9^/L, refer: 10 × 10^9^~26 × 10^9^/L), a high immature/total neutrophil ratio (I/T: 0.14), low platelet count (PLT 25 × 10^9^/L, refer: 150∼300 × 10^9^/L), elevated CRP (29.98 mg/L, refer: 0∼8 mg/L), and procalcitonin (PCT 57.34ng/L, refer: 0∼0.5 ng/L). Blood cultures from peripheral blood and the catheter tip were taken immediately, mechanical ventilation was reintroduced, and empirical antibiotic therapy was escalated to meropenem (0.04 g) and fluconazole (13 mg). Despite aggressive interventions, including acidosis correction, volume expansion, circulatory support, anemia correction, coagulation factor supplementation, continuous cardiopulmonary resuscitation, and epinephrine administration, the patient’s heart rhythm and oxygen saturation fluctuated, and he was declared clinically dead after 7 h and 5 min of resuscitation. Peripheral blood (two bottles) and catheter tip (two bottles) cultures returned positive for *Bacillus cereus* after 14 h. However, due to the child’s death, antimicrobial susceptibility testing was not conducted. Despite extensive therapeutic efforts, the infant succumbed to *Bacillus cereus* infection.

### Case 2

**Fig. 2:**
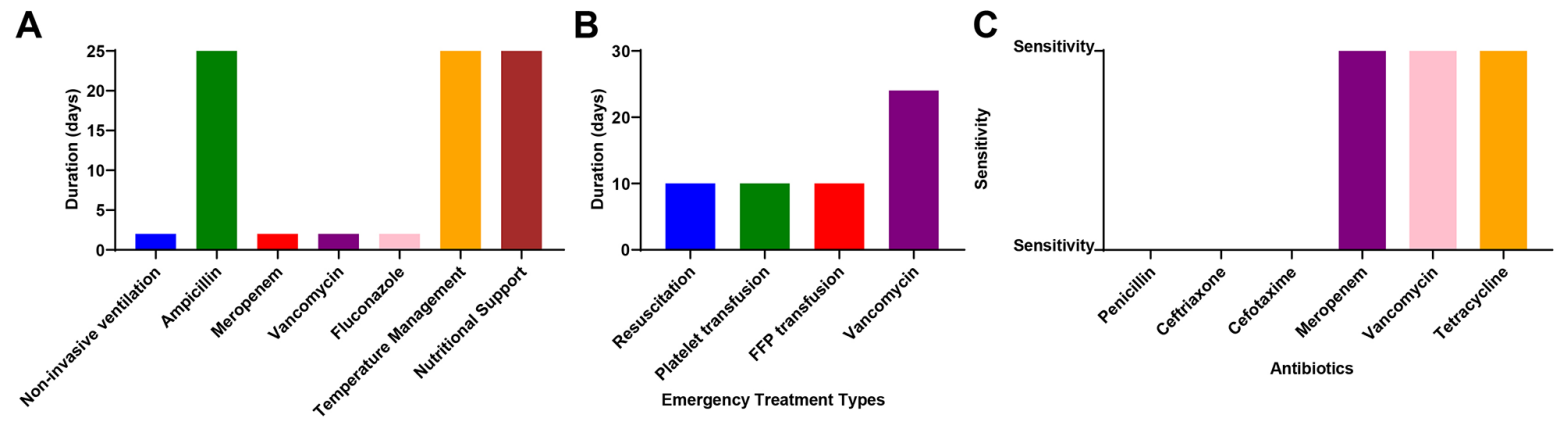
Progressive clinical course and therapeutic interventions in case 2 with *Bacillus cereus* sepsis. Note: **(A)** Detailed description of treatment measures (non-invasive ventilation, antibiotic therapy, temperature, and nutritional maintenance); **(B)** Response to emergencies (resuscitative treatment, transfusions, mechanical ventilation); **(C)** Infection diagnosis and antibiotic sensitivity test results

A female neonate, the first child of a third pregnancy, was born at 28 + 2 weeks gestation, weighing 1030 g. The 21-year-old mother had a history of prolonged premature rupture of membranes exceeding 18 h and urinary tract infection during pregnancy but no record of preeclampsia. The neonate’s Apgar score was 8 at 1 min post-birth, with no record at 5 min. She was admitted to the NICU of a local hospital due to respiratory distress syndrome, prematurity, and meager birth weight. Table 1; Fig. 2 displayed her detailed clinical condition and initial response to treatment upon admission.

**Table 1:** Laboratory results upon admission (coagulation parameters, complete blood count, CRP levels)

	Admission Refernce value range	
APTT(s)	62.1	23∼37
PT(s)	13.7	10∼14.3
Hb(g/L)	164	170∼200
HCT(%)	50.10%	48∼68
WBC(×109 g/L)	30.12	10∼26
PLT(×109 g/L)	276	150∼300
CRP(mg/L)	1.53	0∼8

Upon admission, her vital signs were: temperature 36.6 °C, heart rate 135 beats per minute, and respiratory rate 35 breaths per minute. She appeared lethargic, with bruising on her right dorsal foot. The anterior fontanelle was flat and soft. Pupils responded equally to light. Coarse breath sounds were noted bilaterally without crackles or wheezes. Her heart rhythm was regular at 135 beats per minute, with no murmurs detected. Her abdomen was soft, with the liver palpable 1 cm below the right costal margin and the spleen not palpable. Bowel sounds were normal. Primitive reflexes such as rooting, sucking, and grasping were not elicited. Initial laboratory investigations revealed coagulation parameters: Activated Partial Thromboplastin Time (APTT) 62.1 s (refer: 23–37 s), Prothrombin Time (PT) 13.7 s (refer: 10–14.3 s); complete blood count: Hemoglobin (Hb) 164 g/L (refer: 170–200 g/L), Hematocrit (HCT) 50.10% (refer: 48-68%), WBC 30.12 × 10^9^/L (refer: 10 × 10^9^-26 × 10^9^/L), PLT 276 × 10^9^/L (refer: 150–300 × 109/L), and CRP 1.53 mg/L (refer: 0–8 mg/L). Treatment measures initiated included non-invasive ventilation, prophylactic administration of ampicillin (0.05 g, q12h) for infection prevention, maintenance of body temperature, and intravenous nutrition. Furthermore, on the third day of hospitalization, a peripherally inserted central catheter (PICC line) was inserted.

On the 24th day after birth, the patient displayed fatigue and pallor during nasal catheter oxygen administration, with weakened spontaneous breathing and fluctuating heart rate between 190 and 210 beats per minute. Capillary refill time was noted to be 5 s. The emergency blood gas analysis revealed a pH of 6.985 (refer: 7.35∼7.45), PCO_2_ of 80.4 mmHg (refer: 35∼45 mmHg), lactate level of 9.7 mmol/L (refer: 1.6–2.5 mmol/L), and Base Excess (BE) of -12.4 mmol/L (refer: -6.6∼2.54 mmol/L). Acute CBC results indicated a white cell count of 1.88 × 10^9^/L (refer: 10 × 10^9^~26 × 10^9^/L), neutrophils at 74% (refer: 10 × 10^9^~26 × 10^9^/L), I/T ratio of 0.16, platelet count of 16.00 × 10^9^/L (refer: 150∼300 × 10^9^/L), C-reactive protein level of 64.78 mg/L (refer: 0∼8 mg/L), and procalcitonin level of 99.36 ng/L (refer: 0∼0.5 ng/L). Considering the early signs of septic shock and coagulation dysfunction, the patient underwent resuscitation treatment with normal saline, acid-base balance adjustment, and vasopressor agents. Due to the coagulation dysfunction, platelet transfusion and fresh frozen plasma therapy were administered. Endotracheal intubation and mechanical ventilation were implemented. The PICC catheter was removed, and peripheral blood along with the catheter tip was sent for culture. Empirical antibiotic therapy was initiated consisting of meropenem (0.04 g, every 8 h), vancomycin (18 mg, every 8 h), and fluconazole (14 mg, single dose). Extensive echogenic changes were observed in the emergency cranial ultrasound examination.

On the 25th day of hospitalization, her condition continued to deteriorate. Despite invasive ventilation, the child’s oxygen saturation remained unstable, fluctuating between 70 and 95%. Significant hypotension was observed, with the lowest mean arterial pressure dropping to 15 mmHg. Physical examination revealed anterior fontanelle bulging and pale skin. An urgent bedside cranial ultrasound showed diffuse echogenic changes in brain structures and enhanced echogenicity in the basal ganglia, suggestive of intracerebral hemorrhage. Despite resuscitation efforts, the child’s C-reactive protein (CRP) level escalated to 111.56 mg/L (refer: 0–8 mg/L). Coagulation tests indicated prolonged PT (32.2 s) (refer: 10–14.3 s), TT (20.4 s) (refer: 8–16 s), APTT (83 s) (refer: 23–43 s), and decreased Fibrinogen (FIB) (1.40 g/L) (refer: 2–4 g/L). We utilized epinephrine to constrict vasculature and elevate blood pressure, along with dexamethasone to reduce inflammation. Blood culture and catheter tip cultures performed after 10 and 11 h, respectively, isolated *Bacillus cereus*. The sensitivity analysis showed resistance to penicillin (MIC > 32), ceftriaxone (MIC > 32), cephalothin (MIC > 256), and cefotaxime (not detected), while meropenem (MIC 0.19), vancomycin (MIC < 4), tetracycline (MIC 4), and erythromycin (MIC 0.25) demonstrated sensitivity.

On day 26, the neonate’s overall response significantly declined. Despite invasive ventilation, her oxygen saturation remained unstable, and her blood pressure occasionally dropped. Her condition improved slightly after fluid resuscitation, but peripheral circulation remained poor. She was diagnosed with diffuse cerebral softening and intracranial hemorrhage. Despite maximal supportive care, her condition continued to deteriorate. Given the poor prognosis, the family decided to discontinue treatment, and the infant unfortunately passed away on the following day. Figure 2 provides a detailed course of the illness and treatment measures, including the final clinical outcome and treatment decisions.

## Discussion

Over time, infections of *Bacillus cereus* in neonates have garnered widespread attention in the medical community. To further investigate the characteristics and clinical significance of these infections, we synthesized information from 11 related studies [15–25] and 11 case reports from our institution, summarizing detailed data on 58 neonatal *Bacillus cereus* infections (Table 2).

**Table 2:** Comprehensive Summary of 58 Neonatal Bacillus cereus Infection Cases Derived from 11 Studies and Our Hospital Case Reports

References	Numbers	Birth Weight/Gestational Age	Days	Infection Site	Treatment	Prognosis	Possible Mode of Transmission
Case 1	1	940 g/27weeks^+ 2^ days	23	Blood	Meropenem	Deceased	Undetermined
Case 2	1	1030 g/28weeks^+ 1^ day	25	Blood	Vancomycin, Meropenem	Deceased	Undetermined
Case 3	1	2430 g/34weeks^+ 4^ days	7	Blood/Abdominal/Bacterial Meningitis	Vancomycin, Meropenem, Piperacillin-Tazobactam	Recovered	Undetermined
Case 4	1	1070 g/32weeks^+ 6^ days	24	Blood/Abdominal/Bacterial Meningitis	Vancomycin, Meropenem, Piperacillin-Tazobactam	Recovered	Undetermined
Case 5	1	980 g/29weeks^+ 5^ days	21	Blood	Vancomycin, Meropenem	Recovered	Undetermined
Case 6	1	1940 g/32weeks^+ 5^ days	8	Blood/Brain Abscess	Vancomycin, Meropenem	Recovered	Undetermined
Case 7	1	1880 g/35weeks^+ 2^ days	16	Blood	Vancomycin, Piperacillin-Tazobactam	Recovered	Undetermined
Case 8	1	1710 g/32weeks^+ 3^ days	9	Blood	Vancomycin, Meropenem, Piperacillin-Tazobactam	Recovered	Undetermined
Case 9	1	995 g/30weeks^+ 5^ days	23	Blood/Abdomen	Vancomycin, Meropenem	Deceased	Undetermined
Case 10	1	2320 g/33weeks^+ 2^ days	11	Blood/Purulent Meningitis	Vancomycin	Recovered	Undetermined
Case 11	1	1300 g/30weeks^+ 5^ days	35	Blood/Purulent Meningitis with Multiple Cerebral Softening	Vancomycin, Meropenem	Deceased	Undetermined
Torjesen I [15]	18	N/A	N/A	Blood	N/A	1 case Deceased 17 cases Recovered	Parenteral nutrition
[16]	2	N/A	N/A	Lung	Cefazolin	Deceased	Bed sheet
[16]	2	N/A	N/A	Lung	Meropenem, Vancomycin, Piperacillin	Deceased	Bed sheet
[17]	2	ND/32weeks	6	Blood/Brain abscess	Vancomycin, Meropenem, Chloramphenicol	Deceased	Construction dust
[18]	2	590 g/25weeks	4	Blood	Linezolid, Meropenem, Vancomycin,	Deceased	Milk from milk bank
[18]	2	560 g/24weeks	6	Blood	Tobramycin, Vancomycin, Meropenem, Piperacillin-tazobactam, Fluconazole, Amphotericin B, Compound sulfamethoxazole	Deceased	Milk from milk bank
[19]	1	ND/26weeks^+ 3^	3	Lung (Necrotizing pneumonia)	Amoxicillin, Gentamicin	Deceased	Undetermined
[20]	2	2700 g/37weeks^+ 5^	N/A	Lung	Vancomycin, Meropenem, Amphotericin	Deceased	Undetermined
[21]	2	880 g/27weeks^+ 2^	4	Blood	Cefotaxime, Gentamicin, Vancomycin, Levofloxacin	Deceased	Undetermined
[21]	2	1480 g/29weeks^+ 4^	4	Blood	Cefotaxime, Gentamicin, Vancomycin	Deceased	Undetermined
[22]	1	740 g/27weeks	2	Blood/Central nervous system/Lung	Ceftazidime	Deceased	Undetermined
[17]	2	ND/31weeks	4	Blood	Vancomycin, Meropenem	N/A	Construction dust
[23]	1	800 g/N/A	5	Blood/Central nervous system	Imipenem, Meropenem, Gentamicin, Clindamycin, Vancomycin, Linezolid, Levofloxacin	Recovered	Bath towel
[24]	2	960 g/29weeks	3	Severe necrotizing enterocolitis	Vancomycin, Cefotaxime, Metronidazole	Recovered	Breast milk
[24]	2	1500 g/30weeks	3	Severe necrotizing enterocolitis	Vancomycin, Cefotaxime, Metronidazole	Recovered	Breast milk
[20]	2	815 g/27weeks^+ 1^	17	Lung	Vancomycin, Meropenem,	Recovered	Undetermined
[25]	1	1910 g/32weeks^+ 4^	6	Blood/Brain abscess	Vancomycin, Amikacin, Gentamicin, Meropenem	Recovered	Undetermined

The average birth weight of the 58 neonates in this study was 1306.9 g, with weights ranging from 529 g to 2700 g. Their average gestational age was 28.85 weeks, ranging from 24 weeks to 38 weeks. It is noteworthy that these infections were first detected on average 11.21 days after birth, with durations ranging from 2 days to 35 days. Among these infection cases, the most common abnormalities observed included a significant decrease in white blood cell count (as low as 1.88 × 10^9^/L) and a pronounced prolongation of the prothrombin time in the blood. Additionally, the infants exhibited widespread crackles in the lungs, and in severe cases, respiratory distress syndrome, and symptoms of severe central nervous system infection were present (as indicated by widespread echogenic changes on cranial ultrasound). From the aforementioned conditions, it is apparent that the infections were not merely gastrointestinal but rather severe systemic multi-organ failure caused by Bacillus cereus infection. Consequently, there was a mortality rate as high as 41.38% among these 58 cases, with 24 cases unfortunately resulting in death.

**Fig. 3:**
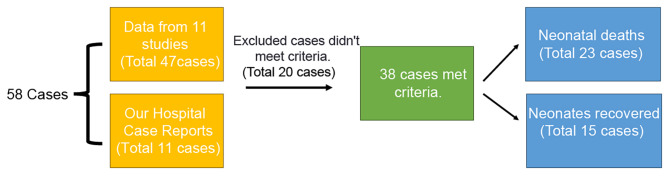
Cases statistics process

**Fig. 4:**
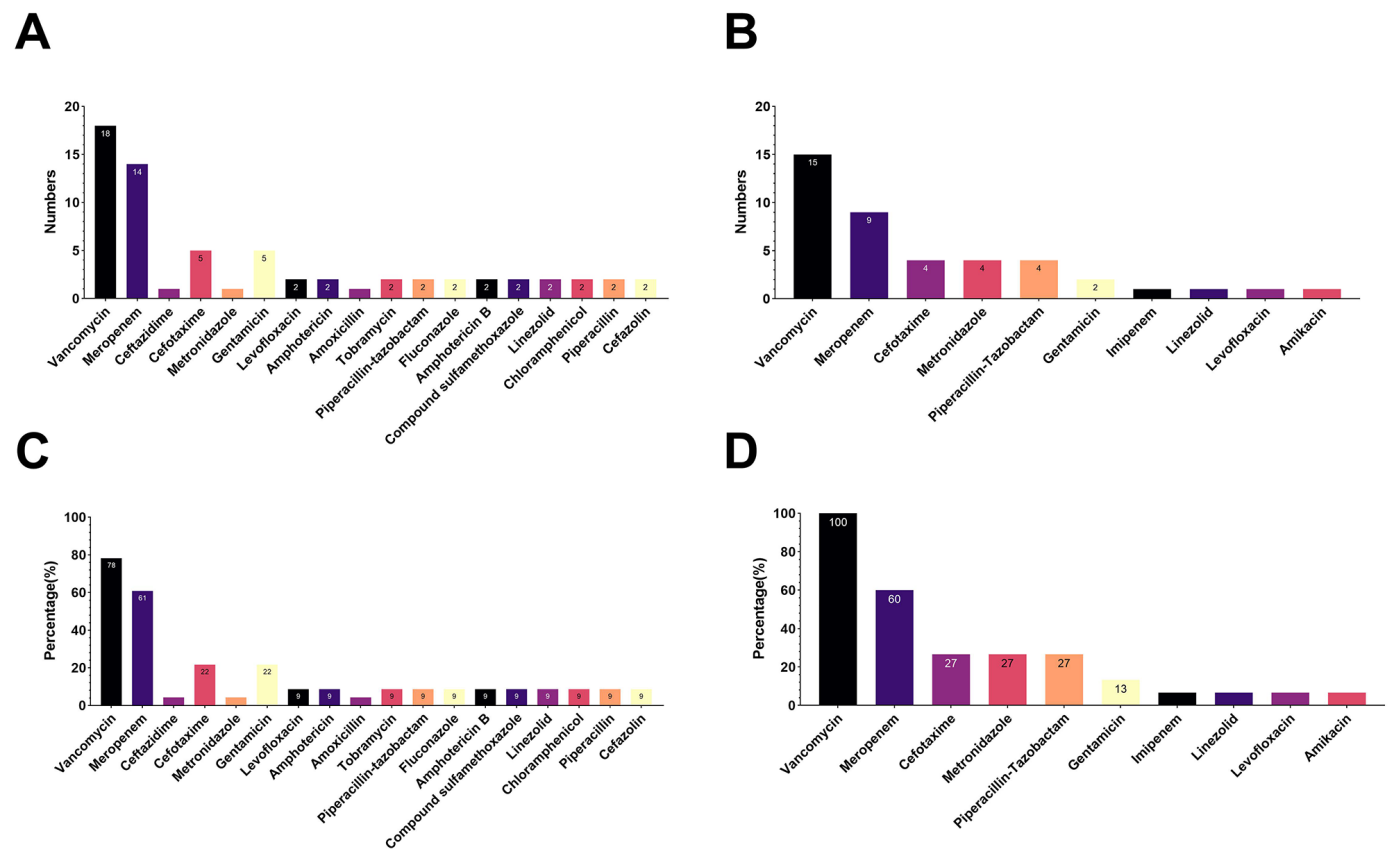
Summary of treatment approaches for neonatal infections caused by *Bacillus cereus*. Note: **(A)** Number of treatments applied in deceased cases (based on 23 cases); **(B)** Percentage of treatments used in deceased cases (based on 23 cases); **(C)** Number of treatments applied in recovered cases (based on 15 cases); **(D)** Percentage of treatments used in recovered cases (based on 15 cases

A further comparative analysis was conducted on cases of death and recovery. After excluding the information provided by Torjesen I [15] and Bar-Meir et al. [17], a total of 38 cases met the inclusion criteria (Fig. 3). Among the 23 cases resulting in death, 7 presented with lung infections and 16 with blood infections. The average birth weight of these neonates was 1067.64 g, with a mean gestational age of 28.4 weeks, and infections were first detected at an average of 12.27 days. Treatment regimens primarily included Vancomycin, Meropenem, Ceftazidime, Cefotaxime, Metronidazole, Gentamicin, Levofloxacin, Amphotericin, Amoxicillin, Tobramycin, Piperacillin-tazobactam, Fluconazole, Amphotericin B, Compound sulfamethoxazole, Linezolid, Chloramphenicol, Piperacillin, and Cefazolin (Fig. 4A). Vancomycin and meropenem were the most frequently used, at 78% and 61%, respectively (Fig. 4B).

Conversely, among the 15 recovered cases, 2 had lung and 9 had blood infections. These neonates had an average birth weight of 1221 g and a mean gestational age of 23.30 weeks, with infections first detected on average 10.83 days post-birth. Treatments mainly involved Vancomycin, Meropenem, Cefotaxime, Metronidazole, Piperacillin-tazobactam, Gentamicin, Imipenem, Linezolid, Levofloxacin, and Amikacin (Fig. 4C). Vancomycin use was 100%, Meropenem 60%, Cefotaxime 27%, Metronidazole 27%, and Piperacillin-tazobactam 27% (Fig. 4D).

From this data, lung infections are more common in deceased cases, suggesting a significant factor in mortality. Furthermore, infections in recovered cases were detected earlier, emphasizing the critical role of early detection and diagnosis in improving recovery rates. The selection of treatment methods also highlights their importance in recovery. The variety of medications used in recovered cases was comparatively less, suggesting targeted medication is a critical factor in improving recovery rates.

Beyond causing food poisoning, *Bacillus cereus* can lead to various non-gastrointestinal or systemic infections in neonates, including sepsis, pneumonia, meningitis, and anthrax-like skin infections, as indicated by Table 2’s literature. The mortality rate of these infection cases is significantly high, with a poor prognosis. In addition, the placement of central venous catheters in neonates, hospital environment, and nutritional formulas are important external factors contributing to neonatal infections. Furthermore, in 2018, India reported 12 cases of severe neonatal skin infections resembling anthrax-like lesions caused by *Bacillus cereus*. These infections originated from vesicle-like or ruptured vesicle-like lesions, accompanied by extensive and rapid spread of cellulitis. Genetic analysis of the skin isolates revealed the genetic relationships among these strains [27]. Another study reported a case of severe pulmonary tissue necrosis in a neonate caused by *Bacillus cereus*, who sadly passed away only 65 h after birth [19]. Clara Machado and colleagues reported a case of neonatal meningitis caused by *Bacillus cereus*. The neonate’s brain tissue showed extensive damage and necrosis, eventually forming a brain abscess. Despite appropriate antibiotic treatment and surgery, the infant survived but with developmental delays. Studies indicate that central nervous system infections caused by *Bacillus cereus* in neonates are likely to result in death [28].

Of the 58 recorded non-gastrointestinal neonatal infections, 24 resulted in death, with a mortality rate of 41.38%. Mari Saito’s research found that the microbial characteristics of *Bacillus cereus* might be closely related to the high mortality rate of these infections. The study discovered that hemolysins and phospholipases produced by *Bacillus cereus* can cause hemolytic changes in neonatal blood and cerebrospinal fluid, potentially leading to symptoms similar to intraventricular hemorrhage [22]. For neonates with catheters or compromised immune systems, this can have fatal consequences. Elrike Frenzel et al. also discovered that *Bacillus cereus* can form slow-growing minor colony variants through phenotypic switching under the influence of aminoglycoside antibiotics [29]. These variants differ significantly from the wild type in development, phenotype, metabolism, and toxicity, often leading to misdiagnosis and antibiotic treatment failure in standard identification tests [29].

In treating non-gastrointestinal infections caused by *Bacillus cereus*, delayed and failed use of antibiotics is a significant factor in adverse outcomes [30]. The study showed that *Bacillus cereus* strains isolated from neonatal blood or the environment were sensitive to vancomycin. According to research by Mahoko Ikeda et al., vancomycin is the optimal choice for treating blood infections caused by *Bacillus cereus* [31]. Anna B. John successfully treated a preterm infant’s persistent *Bacillus cereus* infection with vancomycin [32]. However, since *Bacillus cereus* is widely present in the environment and often regarded as a laboratory contaminant, clinicians may need more timely information. This delay can hinder the prompt selection of effective antibiotics for non-gastrointestinal infections caused by this bacterium in neonates. Thus, when *Bacillus cereus* is isolated from sterile sites in clinical microbiology laboratories, comprehensive judgment based on whether the child has undergone invasive procedures, inflammatory indicators, etc., should be considered, rather than immediately dismissing it as a contaminant, to avoid treatment delays.

Comprehensive infection prevention and control measures are necessary to prevent the spread of *Bacillus cereus* infections in neonatal wards. Utilizing microbiological techniques to determine the genetic relationship between clinical specimens and medical environments or objects can be very effective. For instance, Torjesen’s study identified a batch of intravenous nutritional products contaminated with *Bacillus cereus*, leading to sepsis in 18 neonates. By replacing the nutritional products, the risk of neonatal infection was successfully reduced [15]. George Turabelidze et al. found genetic relationships between *Bacillus cereus* strains isolated from clinical specimens of children and respiratory ventilator sensors. By switching to steam sterilization, the spread of *Bacillus cereus* was successfully halted [33]. Researchers like Shimono collected *Bacillus cereus* strains from instrument surfaces and air. By rigorously cleaning ventilation ducts and covering them to prevent dust accumulation, an outbreak of neonatal bloodstream infections was successfully controlled [34]. However, in practical clinical settings, the source of contamination leading to non-gastrointestinal infections in neonates caused by Bacillus cereus is not always identifiable. Among 11 relevant studies, only 6 utilized various microbiological methods to confirm the disease transmission pathway. Therefore, given the widespread and elusive nature of *Bacillus cereus* transmission, early detection, diagnosis, and appropriate antibiotic therapy are crucial for managing late-onset sepsis in neonates caused by this pathogen.

## Conclusion

In summary, *Bacillus cereus* represents a particularly lethal bacterium for neonates. Its microbiological characteristics, such as the production of pathogenic factors and the ability to transform into minor colony variants, make it a challenging pathogen to treat. While treatment options like vancomycin (18 mg) are viable, the ubiquity of *Bacillus cereus* in the environment and potential laboratory misdiagnoses can lead to treatment delays. Therefore, accurate and timely diagnosis and appropriate treatment strategies are crucial. Effective infection prevention and control measures, including comprehensive strategies, are essential in preventing infections. Overall, a deeper understanding and investigation of *Bacillus cereus* and effective treatment and prevention measures are vital for ensuring neonatal safety.

Although our study consolidates findings from multiple research efforts, the sample size is relatively small, and the transmission pathways of many cases remain unidentified. This literature may not fully represent the true spectrum of neonatal infections. However, with further research and expanded sample collection, we hope to gain a more comprehensive understanding of the characteristics of *Bacillus cereus* in neonatal infections and effective treatment strategies. Ultimately, we remain optimistic about advancements in treatment methods and diagnostic techniques, believing they will address this health threat more effectively.

### Electronic supplementary material

Below is the link to the electronic supplementary material.

Supplementary Material 1

## Data Availability

No datasets were generated or analysed during the current study.
