# Supplementary figures and images for: Late-onset sepsis in newborns caused by Bacillus Cereus: a case report and literature review
Source: Ann Clin Microbiol Antimicrob. 2024 Jul 26;23:66. doi: 10.1186/s12941-024-00712-4 (PMC11282708; doi:10.1186/s12941-024-00712-4)

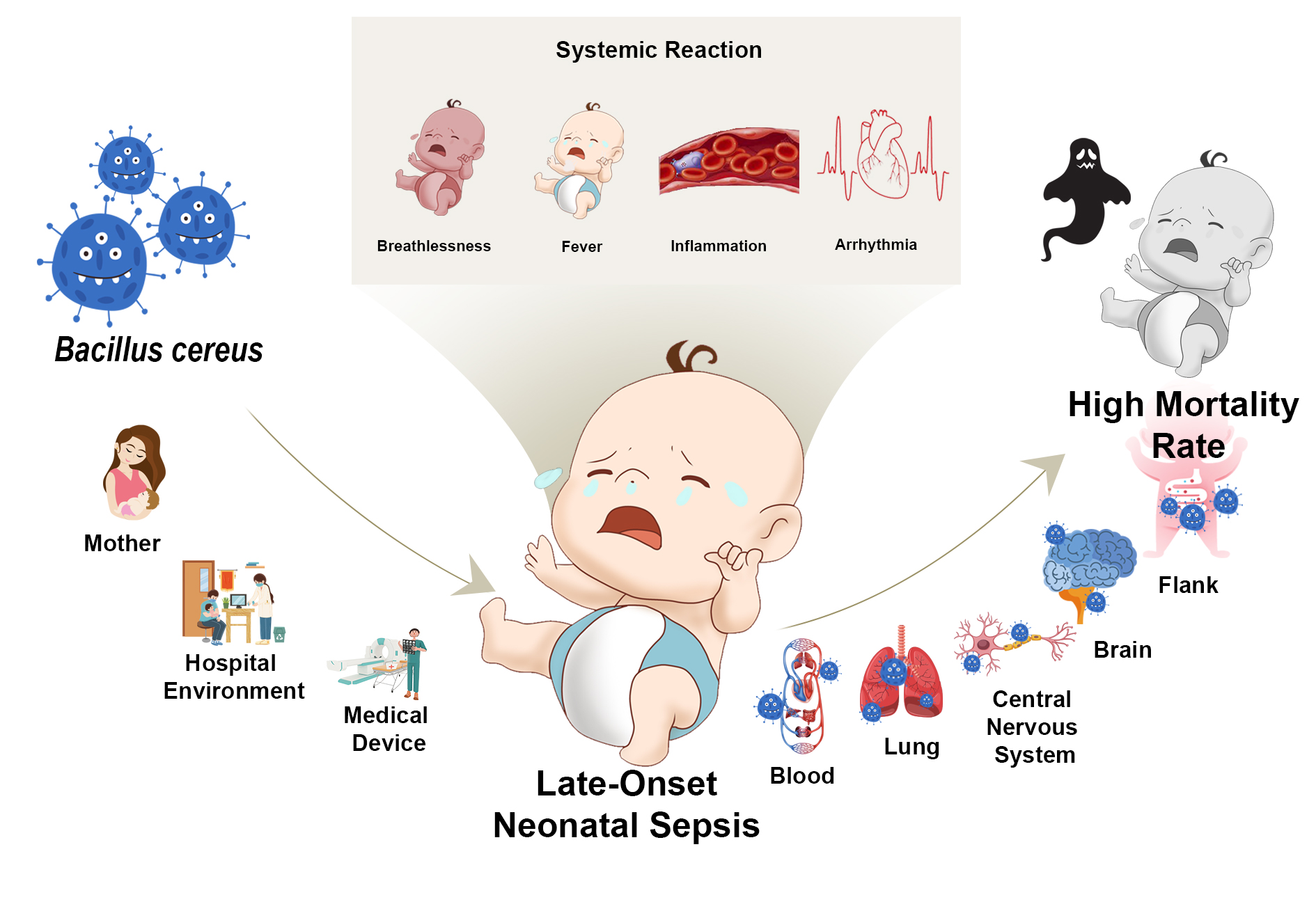

Supplement: Supplementary file 1 — Supplementary Material 1 [file 12941_2024_712_MOESM1_ESM.jpg]
